# Factors affecting well-being among retired older adults: A protocol for an umbrella review

**DOI:** 10.1371/journal.pone.0351425

**Published:** 2026-06-17

**Authors:** Yasin M. Yasin, Areej Al-Hamad, Kateryna Metersky, Emily Read, Richelle Witherspoon

**Affiliations:** 1 Faculty of Nursing and Health Sciences, University of New Brunswick, New Brunswick, Canada; 2 Daphne Cockwell School of Nursing, Faculty of Community Services, Toronto Metropolitan University, Toronto, Canada; 3 Information Services, University of New Brunswick, Fredericton, New Brunswick, Canada; Lingnan Normal University, CHINA

## Abstract

**Background:**

As global populations age, retirement has emerged as a critical life transition influencing well-being across physical, mental, social, financial, and spiritual domains. Although numerous systematic reviews have examined aspects of well-being in retirement, the evidence remains fragmented, with limited integration across domains. No umbrella review has comprehensively synthesized determinants and interventions influencing multidimensional well-being among retired older adults.

**Methods:**

This protocol describes an umbrella review that will synthesize evidence from systematic reviews examining factors and policy, programmatic, community-based, or other supportive efforts associated with physical, mental, social, financial, and spiritual well-being among retired older adults. The review follows Joanna Briggs Institute (JBI) methodology for umbrella reviews and is reported in accordance with PRISMA-P guidance. The search strategy was developed in consultation with a knowledge synthesis librarian and peer reviewed using the PRESS guidelines. Database searches were conducted in MEDLINE (Ovid), CINAHL, PsycINFO, Scopus, and the Cochrane Database of Systematic Reviews for articles published from 2015 onward. Backward and forward citation searching will be undertaken. Two reviewers will independently screen records, extract data, and appraise methodological quality using the JBI Critical Appraisal Checklist for Systematic Reviews and Research Syntheses. Findings will be synthesized narratively. This protocol has been registered with PROSPERO (CRD420251158970).

**Discussion:**

This umbrella review will provide an integrated, high-level synthesis of existing evidence on well-being in retirement, informing future research, policy, and practice aimed at supporting holistic and equitable aging.

## Introduction

Research on the factors influencing well-being among retired older adults has become increasingly important in the context of global population aging. Rising life expectancy has resulted in a demographic shift with older adults now representing one of the fastest-growing population groups worldwide. By 2030, the number of individuals aged 60 years and older is expected to exceed 1.4 billion globally [1]. The population aged 65 years and older has increased substantially over recent decades, rising from approximately 260 million in 1980 to over 760 million in the early 2020s, and is projected to reach nearly 1.5 billion by 2050 [1]. These unprecedented demographic shifts intensify the need to understand the determinants of well-being in later life, particularly during retirement, which is a major life transition that can significantly shape physical, mental, social, financial, and spiritual outcomes.

Retirement is a multidimensional transition that can influence well-being both positively and negatively. Some older adults experience improved life satisfaction, reduced work-related stress, and greater autonomy following retirement [2]. Others, particularly those retiring involuntarily or earlier than planned, have heightened risks of depression, anxiety, social isolation, and loss of identity [3–5]. Physical well-being also fluctuates in retirement, with some individuals adopting healthier routines and increased physical activity [6], while others experience increased sedentary behavior and greater vulnerability to chronic disease [7,8]. Evidence additionally suggests that retirement may be associated with cognitive changes, including declines in executive function [9].

Social well-being similarly undergoes important changes. For some, the loss of work-based networks leads to reduced social interaction, particularly among men who depend heavily on workplace connections [10,11]. For others, retirement provides opportunities to strengthen familial relationships, engage in social and community activities, or participate in volunteer work, all of which can enhance social connectedness and purpose [12].

Financial well-being remains one of the most influential predictors of retirement quality, as many retirees transition from employment income to using pensions or accessing their savings. In Canada, a substantial proportion of future retirees are projected to experience a decline in their standard of living after retirement, with estimates suggesting that between 17% and 50% may face financial shortfalls depending on income level, savings, and pension coverage [13]. These risks are not evenly distributed, as Canadian women face heightened financial vulnerability in retirement due to persistent gender-based income gaps, interrupted employment trajectories, and lower lifetime pension accumulation [14].

Spiritual well-being—though less frequently studied—plays an important role in aging and retirement. Greater spiritual engagement and religious involvement have been shown to enhance psychological resilience, reduce depression, and strengthen coping and ego integrity in older adults [15–17]. Spiritual practices may help retirees navigate existential challenges and reappraise life meaning during later life transitions.

Although a substantial body of literature has examined well-being in later life, the existing evidence remains fragmented and limited in scope. Many interventions designed to improve well-being in older adults focus on a single lifestyle behavior, and most fail to integrate the broader social determinants of health that shape quality of life during retirement [18]. Systematic reviews typically address only one or a few dimensions of well-being, such as mental health [19,20], social participation [21], financial preparedness [22], or physical health [23]. Few attempts have been made to synthesize these domains collectively in a way that reflects the multidimensional reality of well-being among retirees. Existing reviews also demonstrate important limitations in their scope and focus. For example, the systematic review by Teques, Carreiro [24] examined retirement and well-being only within European contexts, which restricts its applicability to diverse global populations. Other reviews have explored predictors of retirement experiences without concentrating specifically on older adults [25]. One umbrella review was located that examined the impact of retirement on physical and mental well-being, with subgroup differences based on financial status, but it did not focus on the older adult population specifically and omitted the social and spiritual dimensions of well-being that are central to holistic aging [26].

Taken together, this evidence highlights a clear gap. Older adults’ well-being in retirement is shaped by complex and interconnected factors across physical, mental, social, financial, and spiritual domains, yet no existing umbrella review provides a comprehensive synthesis across all five determinant domains. An umbrella review is especially suited to addressing this gap because it allows findings from multiple systematic reviews to be compared, appraised, and synthesized at a higher level. This approach can make visible cross-domain patterns, areas of convergence and divergence, methodological limitations, and neglected dimensions of well-being that may not be apparent within single-domain reviews. A high-level, integrated review is needed to consolidate existing systematic reviews, identify cross-domain influences, highlight neglected determinants, and guide the development of more holistic policies and interventions that support older adults during retirement.

To address these gaps, the present umbrella review aims to answer the following research questions:

1. What factors influencing physical, mental, social, financial, and spiritual well-being among retired older adults have been reported in existing systematic reviews?
2. What interventions aimed at improving physical, mental, social, financial, and spiritual well-being among retired older adults have been examined in existing systematic reviews?

## Materials and methods

An umbrella review is a form of evidence synthesis that systematically identifies, appraises, and synthesizes findings from existing systematic reviews rather than from individual primary studies [27]. Unlike a standard systematic review, which typically synthesizes primary research on a focused question, an umbrella review provides a higher-level synthesis of evidence across multiple systematic reviews addressing related questions [28]. It also differs from a scoping review, which is primarily designed to map the breadth and characteristics of a body of literature and may include diverse types of evidence without necessarily appraising methodological quality [29]. Given that several systematic reviews have examined specific aspects of retirement and well-being, an umbrella review is appropriate for the present study because it enables comparison and integration of review-level evidence across multiple well-being domains.

This umbrella review will be conducted in accordance with the JBI methodology for umbrella reviews, which provides a structured and rigorous approach for synthesizing evidence from existing systematic reviews [27]. The review protocol has been prospectively registered with the International Prospective Register of Systematic Reviews (PROSPERO#: CRD420251158970). The methods outlined in this protocol will follow the guidance provided by JBI for review question formulation, eligibility criteria, critical appraisal, data extraction, and synthesis to ensure transparency, methodological consistency, and reproducibility. This protocol was prepared in accordance with the Preferred Reporting Items for Systematic Review and Meta-Analysis Protocols (PRISMA-P) statement [30]. See Appendix 1 for PRISMA-P checklist.

At the time of submission of this protocol, database searches have been completed in September 2025. Title and abstract screening are expected to be completed by end of February 2026, full-text screening by April 2026, and data extraction and quality appraisal by May 2026. Data synthesis and manuscript preparation are anticipated to be completed by July 2026, with results expected to be submitted for publication by August 2026. Any deviations from this timeline will be documented and reported in the final manuscript.

### Inclusion criteria

#### Population.

This umbrella review will include systematic reviews that examine retired older adults, defined in this review as individuals who have exited paid employment and are described by the review authors as retired, in retirement, or post-retirement. Reviews will be eligible if they focus on populations typically aged 50 years and older, a threshold commonly used in international aging and retirement research to enhance comparability across countries with differing retirement policies, labor market structures, and life expectancy. Notably, global aging research initiatives such as the WHO’s Study on Global AGEing and Adult Health (SAGE) define and sample older adult populations beginning at age 50, providing an established precedent for cross-national synthesis of health and well-being in later life [31].

Because retirement is shaped by institutional, cultural, and policy contexts, this review will not require a uniform statutory retirement age across countries. Instead, reviews will be included when retirement status is explicitly identified by the review authors or when retirement is treated as a central exposure, transition, or population characteristic. Reviews that include broader adult populations will be considered eligible only when retirement is an explicit focus and when findings are clearly applicable to retired older adults, such as through subgroup analyses, stratified reporting, or interpretation specific to retirees. Reviews that focus exclusively on older adults without reference to retirement status will not be included unless retirement is explicitly addressed as a key characteristic or exposure.

Country-level demographic indicators, such as fertility or mortality thresholds, will not be used as eligibility criteria because the purpose of this review is to synthesize evidence on retired older adults rather than to classify aging societies. However, where available, contextual information such as geographic region, retirement policy context, retirement definitions, and population characteristics will be extracted and described to support interpretation of cross-national differences.

#### Interventions and exposures.

Eligible systematic reviews may examine a broad range of factors, determinants, correlates, predictors, barriers, facilitators, or exposures that influence well-being during retirement. Reviews will be included if they investigate influences operating at individual, interpersonal, community, organizational, or societal levels and explicitly link these influences to well-being outcomes among retired older adults. Reviews addressing contextual or structural influences will be eligible when such influences are clearly situated within the retirement experience and related to one or more dimensions of well-being. Systematic reviews examining interventions aimed at improving well-being during or after retirement will also be eligible. For the purpose of this review, interventions will be interpreted broadly to include policy, programmatic, organizational, community-based, culturally situated, and other supportive efforts designed to influence well-being outcomes among retired older adults. This broader interpretation acknowledges that efforts to support well-being in retirement may vary across national, cultural, and policy contexts and may not always be described using the term “intervention” in the included reviews.

#### Outcomes.

Systematic reviews will be eligible if they report on outcomes related to well-being among retired older adults in at least one of the following domains: physical, mental, social, financial, or spiritual well-being. Reviews that focus primarily on clinical or disease-specific outcomes will only be included if those outcomes are explicitly linked to one or more well-being domains or broader quality-of-life constructs relevant to retirement.

#### Types of studies.

This umbrella review will include systematic reviews, with or without meta-analysis, that synthesize evidence relevant to the review questions. Reviews will be considered “systematic” if they report a clearly stated review objective or question, describe a reproducible search strategy, specify explicit eligibility criteria, and outline a structured approach to study selection and evidence synthesis [32]. Reviews will be eligible regardless of whether they synthesize quantitative, qualitative, or mixed-methods primary studies, provided the review methods meet the criteria above. Publications that do not meet systematic review methodological standards, including non-systematic literature reviews and protocol-only papers without results, will be excluded.

### Search strategy

The development of the search strategy was undertaken in consultation with a knowledge synthesis librarian (RW), whose expertise supported the systematic identification of relevant terminology, controlled vocabulary, database-specific syntax, and search translation across platforms. This expertise was complemented by the subject-matter expertise of the research team in aging, retirement, population health, wellbeing, and qualitative and mixed-methods research. The team contributed to defining the conceptual scope of the review, including the five well-being domains and the retirement-focused population criteria. Together, this combination of information science and content expertise was used to strengthen both the sensitivity and relevance of the search strategy.

The search was conducted in several iterative stages following JBI guidance. First, an exploratory search was performed in CINAHL (EBSCO), and the titles, abstracts, and subject headings of relevant articles were examined to identify key text words and indexing terms related to retirement, older adults, and multidimensional well-being across the five key domains. Second, these preliminary search terms were tested in CINAHL (EBSCO) using various combinations and search fields to refine the strategy and ensure that retrieved results accurately reflected the scope of research on this topic while minimizing irrelevant records. Given the population of interest, the search was limited to articles published from 2015 to the present to capture contemporary conceptualizations of retirement and well-being.

At this stage, the draft search strategy was peer reviewed by a second librarian expert using the Peer Review of Electronic Search Strategies (PRESS) guidelines [33]. PRESS review is a recognized process for improving the quality, comprehensiveness, and reproducibility of electronic search strategies. All recommended modifications were incorporated, and the final strategy was then adapted for each database. The finalized searches were implemented in September 2025 across five databases selected for their relevance to gerontology, health sciences, and social sciences: CINAHL with Full Text (EBSCOhost), PsycINFO, MEDLINE (Ovid), the Cochrane Library, and Scopus (Elsevier) (see Appendix 2 for the full search strategy). All retrieved records have been imported into Covidence systematic review data management software for screening. In addition to database searching, backward and forward citation searching will be conducted to identify any additional eligible reviews from the final selected reviews. Reference lists of all included articles will be screened for studies missed through the databases search, and forward citation tracking will be carried out using Google Scholar.

### Study selection

As of the time of protocol submission, records have been imported into Covidence and duplicate removal has been completed; screening has not yet begun. Two reviewers will independently screen titles and abstracts within Covidence against the predefined inclusion criteria. Full-text articles will then be retrieved and independently assessed by the same reviewers to determine final eligibility. Any disagreements at either stage of screening will be resolved through discussion, and if consensus cannot be reached, a third reviewer will be consulted. The study selection process will be documented using a PRISMA flow diagram, detailing the number of records identified, screened, assessed for eligibility, and included in the umbrella review, as well as reasons for exclusion at the full-text stage.

### Methodological quality assessment

The methodological quality of all included systematic reviews will be independently assessed by two reviewers using the JBI Critical Appraisal Checklist for Systematic Reviews and Research Syntheses [27]. This tool evaluates key aspects of review quality, including the clarity of the review question, appropriateness of the search strategy, adequacy of inclusion criteria, rigor of critical appraisal of primary studies, methods used for data synthesis, and consideration of publication bias.

Each included review will be appraised against the checklist criteria, and judgments will be recorded systematically. Any disagreements between reviewers will be resolved through discussion, and if consensus cannot be reached, a third reviewer will be consulted. The results of the quality appraisal will be reported descriptively in tabular form and will be used to contextualize the findings of the umbrella review rather than to exclude reviews based solely on quality. Methodological quality will be considered during interpretation of the evidence, particularly when summarizing areas of consistency, uncertainty, or divergence across reviews.

### Data collection

Data extraction has not yet commenced and will begin following completion of study screening. Data will be extracted from all included systematic reviews using a standardized data extraction form developed in accordance with JBI guidance for umbrella reviews [27]. The extraction form will be pilot tested by two reviewers on a small number of included reviews and refined as needed to ensure clarity and consistency. Two reviewers will independently extract data, and discrepancies will be resolved through discussion or by consultation with a third reviewer. Extracted information will include bibliographic details, review objectives, population characteristics, review scope, well-being domains addressed, types of factors or interventions examined, key findings relevant to the review questions, and authors’ conclusions. Information related to methodological characteristics of the reviews, including the number and type of primary studies included, will also be recorded to support interpretation of the synthesized evidence. Data extraction will be managed within Covidence, and extracted data will be exported for synthesis and reporting. Any amendments to the data extraction process will be documented and reported transparently in the final manuscript.

### Data summary

Data synthesis will be conducted using a narrative and descriptive approach, as recommended for umbrella reviews [27]. Findings from included systematic reviews will be synthesized at the review level rather than the level of individual primary studies. Extracted data will be organized according to the five predefined well-being domains: physical, mental, social, financial, and spiritual well-being. For each domain, findings will be summarized to describe the types of factors and interventions examined, the direction and consistency of reported associations, and areas of convergence or divergence across reviews.

Tabular summaries will be used to present key characteristics of included reviews, including well-being domains addressed, types of evidence synthesized, and overall conclusions as per JBI recommendation [27]. The results of the methodological quality assessment will be integrated into the synthesis to contextualize the strength and reliability of the evidence, but reviews will not be excluded from synthesis based solely on quality appraisal results. The synthesis will emphasize a structured, transparent comparison of evidence across domains to identify knowledge gaps, underexplored areas, and implications for future research, policy, and practice related to well-being in retirement.

## Discussion

Well-being in retirement is shaped by a complex interplay of physical, mental, social, financial, and spiritual factors [34]. As global populations age and increasing numbers of individuals transition out of the workforce, there is a growing need for evidence that captures the multidimensional nature of well-being in later life [34]. Although a substantial body of research has examined aspects of retirement and aging, the existing evidence base remains fragmented, with systematic reviews typically focusing on single domains or narrow outcomes. This protocol outlines an umbrella review designed to address this fragmentation by providing a comprehensive synthesis of existing systematic reviews across five key dimensions of well-being among retired older adults.

By consolidating evidence from multiple systematic reviews, this umbrella review will offer a high-level overview of what is currently known about the factors and interventions associated with well-being in retirement. Importantly, the review will not only summarize findings within individual domains but will also examine how different domains intersect and are addressed across the literature [27,35]. This integrated perspective is particularly valuable for informing holistic approaches to healthy aging, which require attention to social determinants, financial security, psychological resilience, and meaning-making alongside physical health [34].

The findings of this umbrella review are expected to have implications for research, policy, and practice. For researchers, the review will identify gaps in the existing synthesis literature, highlight underexplored domains, particularly those related to social and spiritual well-being, and inform priorities for future primary studies and systematic reviews. For policymakers and practitioners, the review will provide an evidence-informed foundation to support the development of retirement policies, age-friendly initiatives, and interventions that reflect the multidimensional realities of retirement rather than focusing on isolated outcomes.

From a methodological perspective, this umbrella review is subject to inherent limitations associated with its design. As a synthesis of systematic reviews, the findings will depend on the quality, scope, and reporting of the included reviews and the primary studies they synthesize. Variability in definitions and measurement of well-being across reviews, as well as overlap of primary studies, may limit comparability and influence interpretation of the evidence. These limitations will be addressed through systematic quality appraisal, transparent reporting, and careful contextualization of findings.

The results of this umbrella review will be disseminated through publication in a peer-reviewed open-access journal and presentations at academic and policy-oriented conferences. Findings will also be shared with relevant stakeholders, including researchers, policymakers, and community organizations working in aging and retirement, to support knowledge translation and evidence-informed decision-making.

Any amendments to this protocol, including changes to eligibility criteria, search strategy, or methods, will be documented, dated, and justified in the final publication. If the review is terminated prematurely, the reasons for termination will be clearly reported to ensure transparency and accountability.

## Conclusion

This protocol outlines a rigorous umbrella review designed to synthesize existing systematic reviews on factors and interventions influencing physical, mental, social, financial, and spiritual well-being among retired older adults. By applying JBI methodology and transparent reporting standards, the review will address fragmentation in the current evidence base and provide an integrated, high-level understanding of well-being in retirement. The findings are expected to inform future research, policy, and practice by supporting more holistic, evidence-informed approaches to promoting well-being and quality of life in older retirees.

## Supporting information

S1 Appendix PRISMA-P checklist. (DOCX)

S2 Appendix Search strategy. (DOCX)
