# Supplementary material for: Factors affecting well-being among retired older adults: A protocol for an umbrella review
Source: PLoS One. 2026 Jun 17;21(6):e0351425. doi: 10.1371/journal.pone.0351425 (PMC13274834; doi:10.1371/journal.pone.0351425)
Supplement: S2 Appendix — (DOCX) [file pone.0351425.s002.docx]

# Appendix 2 : Search Strategy

## CINAHL

| **S#** | **Query (user-entered)** | **Query (expanded/display term)** | **Search run Date and Time** | **Results (count)** |
| --- | --- | --- | --- | --- |
| **S43** | S17 AND S39 AND S42 | ((XB (sexagenarian* OR septuagenarian* OR septagenarian* OR octogenarian* OR nonagenarian* OR centenarian* OR centarian* OR supercentenarian* OR supercentarian*)) OR (XB ("65+" OR "65 and over" OR "65 and older" OR "over 65" OR "older than 65")) OR (XB ("60+" OR "60 and over" OR "60 and older" OR "over 60" OR "older than 60")) OR (XB ("55+" OR "55 and over" OR "55 and older" OR "over 55" OR "older than 55")) OR (XB ("50+" OR "50 and over" OR "50 and older" OR "over 50" OR "older than 50")) OR (XB (pensioner*)) OR (XB ("old* person")) OR (XB (late* N2 life)) OR (XB (geriatr*)) OR (XB ("elder*")) OR (XB ("very old")) OR (XB ("oldest old")) OR (XB ("old age")) OR (XB ("older adult*")) OR (XB (senior*)) OR (XB (retired OR retirement OR retiree))) AND ((XB ("aging well")) OR (XB (fulfillment)) OR (XB ("fulfilled life")) OR (XB (meaning* N2 life)) OR (XB ("sense of purpose*")) OR (XB ("optimal aging")) OR (XB (flourish*)) OR (XB (thriving OR thrive*)) OR (XB ("active aging")) OR (XB ("positive aging")) OR (XB ("healthy aging")) OR (XB (happy OR happiness)) OR (XB ("good health")) OR (XB ("mental health")) OR (XB ("cognitive health")) OR (XB ("physical health")) OR (XB ("successful aging")) OR (XB ("life satisfaction")) OR (XB ("quality of life")) OR (XB (wellness)) OR (XB ("well being" OR wellbeing))) AND ((XB ("meta analys*" OR metaanalys* OR "meta synthes*" OR metasynthes*)) OR (XB "systematic review*")) | 2025-09-03T12:14:18.623Z | 2520 |
| **S42** | S41 OR S40 | (XB ("meta analys*" OR metaanalys* OR "meta synthes*" OR metasynthes*)) OR (XB "systematic review*") | 2025-09-03T12:11:19.193Z | 228542 |
| **S41** | XB ("meta analys*" OR metaanalys* OR "meta synthes*" OR metasynthes*) | XB ("meta analys*" OR metaanalys* OR "meta synthes*" OR metasynthes*) | 2025-09-03T12:10:14.515Z | 134778 |
| **S40** | XB "systematic review*" | XB "systematic review*" | 2025-09-03T12:09:40.533Z | 177617 |
| **S39** | S38 OR S37 OR S36 OR S35 OR S34 OR S33 OR S32 OR S31 OR S30 OR S29 OR S28 OR S27 OR S26 OR S25 OR S24 OR S23 OR S22 OR S21 OR S20 OR S19 OR S18 | (XB ("aging well")) OR (XB (fulfillment)) OR (XB ("fulfilled life")) OR (XB (meaning* N2 life)) OR (XB ("sense of purpose*")) OR (XB ("optimal aging")) OR (XB (flourish*)) OR (XB (thriving OR thrive*)) OR (XB ("active aging")) OR (XB ("positive aging")) OR (XB ("healthy aging")) OR (XB (happy OR happiness)) OR (XB ("good health")) OR (XB ("mental health")) OR (XB ("cognitive health")) OR (XB ("physical health")) OR (XB ("successful aging")) OR (XB ("life satisfaction")) OR (XB ("quality of life")) OR (XB (wellness)) OR (XB ("well being" OR wellbeing)) | 2025-09-03T12:04:38.947Z | 436467 |
| **S38** | XB ("aging well") | XB ("aging well") | 2025-09-03T12:03:09.859Z | 238 |
| **S37** | XB (fulfillment) | XB (fulfillment) | 2025-09-03T12:02:53.672Z | 2268 |
| **S36** | XB ("fulfilled life") | XB ("fulfilled life") | 2025-09-03T12:02:38.603Z | 19 |
| **S35** | XB (meaning* N2 life) | XB (meaning* N2 life) | 2025-09-03T12:01:42.332Z | 3739 |
| **S34** | XB ("sense of purpose*") | XB ("sense of purpose*") | 2025-09-03T12:01:22.578Z | 689 |
| **S33** | XB ("optimal aging") | XB ("optimal aging") | 2025-09-03T12:01:14.123Z | 51 |
| **S32** | XB (flourish*) | XB (flourish*) | 2025-09-03T12:01:02.433Z | 2491 |
| **S31** | XB (thriving OR thrive*) | XB (thriving OR thrive*) | 2025-09-03T12:00:55.036Z | 5812 |
| **S30** | XB ("active aging") | XB ("active aging") | 2025-09-03T12:00:40.746Z | 507 |
| **S29** | XB ("positive aging") | XB ("positive aging") | 2025-09-03T12:00:33.216Z | 127 |
| **S28** | XB ("healthy aging") | XB ("healthy aging") | 2025-09-03T12:00:26.099Z | 3396 |
| **S27** | XB (happy OR happiness) | XB (happy OR happiness) | 2025-09-03T12:00:16.893Z | 11548 |
| **S26** | XB ("good health") | XB ("good health") | 2025-09-03T12:00:06.788Z | 4310 |
| **S25** | XB ("mental health") | XB ("mental health") | 2025-09-03T12:00:01.547Z | 156150 |
| **S24** | XB ("cognitive health") | XB ("cognitive health") | 2025-09-03T11:59:53.760Z | 1386 |
| **S23** | XB ("physical health") | XB ("physical health") | 2025-09-03T11:59:44.956Z | 18446 |
| **S22** | XB ("successful aging") | XB ("successful aging") | 2025-09-03T11:59:34.483Z | 1573 |
| **S21** | XB ("life satisfaction") | XB ("life satisfaction") | 2025-09-03T11:59:25.423Z | 7594 |
| **S20** | XB ("quality of life") | XB ("quality of life") | 2025-09-03T11:59:15.856Z | 172104 |
| **S19** | XB (wellness) | XB (wellness) | 2025-09-03T11:59:05.728Z | 265990 |
| **S18** | XB ("well being" OR wellbeing) | XB ("well being" OR wellbeing) | 2025-09-03T11:58:56.418Z | 265990 |
| **S17** | S16 OR S15 OR S14 OR S13 OR S12 OR S11 OR S10 OR S9 OR S8 OR S7 OR S6 OR S5 OR S4 OR S3 OR S2 OR S1 | (XB (sexagenarian* OR septuagenarian* OR septagenarian* OR octogenarian* OR nonagenarian* OR centenarian* OR centarian* OR supercentenarian* OR supercentarian*)) OR (XB ("65+" OR "65 and over" OR "65 and older" OR "over 65" OR "older than 65")) OR (XB ("60+" OR "60 and over" OR "60 and older" OR "over 60" OR "older than 60")) OR (XB ("55+" OR "55 and over" OR "55 and older" OR "over 55" OR "older than 55")) OR (XB ("50+" OR "50 and over" OR "50 and older" OR "over 50" OR "older than 50")) OR (XB (pensioner*)) OR (XB ("old* person")) OR (XB (late* N2 life)) OR (XB (geriatr*)) OR (XB ("elder*")) OR (XB ("very old")) OR (XB ("oldest old")) OR (XB ("old age")) OR (XB ("older adult*")) OR (XB (senior*)) OR (XB (retired OR retirement OR retiree)) | 2025-09-03T11:58:19.901Z | 555422 |
| **S16** | XB (sexagenarian* OR septuagenarian* OR septagenarian* OR octogenarian* OR nonagenarian* OR centenarian* OR centarian* OR supercentenarian* OR supercentarian*) | XB (sexagenarian* OR septuagenarian* OR septagenarian* OR octogenarian* OR nonagenarian* OR centenarian* OR centarian* OR supercentenarian* OR supercentarian*) | 2025-09-03T11:57:28.576Z | 2876 |
| **S15** | XB ("65+" OR "65 and over" OR "65 and older" OR "over 65" OR "older than 65") | XB ("65+" OR "65 and over" OR "65 and older" OR "over 65" OR "older than 65") | 2025-09-03T11:56:22.397Z | 72222 |
| **S14** | XB ("60+" OR "60 and over" OR "60 and older" OR "over 60" OR "older than 60") | XB ("60+" OR "60 and over" OR "60 and older" OR "over 60" OR "older than 60") | 2025-09-03T11:56:05.922Z | 109115 |
| **S13** | XB ("55+" OR "55 and over" OR "55 and older" OR "over 55" OR "older than 55") | XB ("55+" OR "55 and over" OR "55 and older" OR "over 55" OR "older than 55") | 2025-09-03T11:55:46.578Z | 39113 |
| **S12** | XB ("50+" OR "50 and over" OR "50 and older" OR "over 50" OR "older than 50") | XB ("50+" OR "50 and over" OR "50 and older" OR "over 50" OR "older than 50") | 2025-09-03T11:55:30.315Z | 123958 |
| **S11** | XB (pensioner*) | XB (pensioner*) | 2025-09-03T11:54:12.765Z | 324 |
| **S10** | XB ("old* person") | XB ("old* person") | 2025-09-03T11:54:00.425Z | 1751 |
| **S9** | XB (late* N2 life) | XB (late* N2 life) | 2025-09-03T11:53:43.425Z | 17053 |
| **S8** | XB (geriatr*) | XB (geriatr*) | 2025-09-03T11:53:28.563Z | 35309 |
| **S7** | XB ("elder*") | XB ("elder*") | 2025-09-03T11:53:11.543Z | 120346 |
| **S6** | XB ("very old") | XB ("very old") | 2025-09-03T11:52:56.922Z | 1727 |
| **S5** | XB ("oldest old") | XB ("oldest old") | 2025-09-03T11:52:14.258Z | 1939 |
| **S4** | XB ("old age") | XB ("old age") | 2025-09-03T11:51:58.516Z | 9232 |
| **S3** | XB ("older adult*") | XB ("older adult*") | 2025-09-03T11:51:52.125Z | 94226 |
| **S2** | XB (senior*) | XB (senior*) | 2025-09-03T11:51:41.663Z | 29125 |
| **S1** | XB (retired OR retirement OR retiree) | XB (retired OR retirement OR retiree) | 2025-09-03T11:51:15.247Z | 15471 |

## PsycINFO

| **S#** | **Query (user-entered)** | **Query (expanded/display term)** | **Search run Date and Time** | **Results (count)** |
| --- | --- | --- | --- | --- |
| **S43** | S17 AND S39 AND S42 | ((XB (sexagenarian* OR septuagenarian* OR septagenarian* OR octogenarian* OR nonagenarian* OR centenarian* OR centarian* OR supercentenarian* OR supercentarian*)) OR (XB ("65+" OR "65 and over" OR "65 and older" OR "over 65" OR "older than 65")) OR (XB ("60+" OR "60 and over" OR "60 and older" OR "over 60" OR "older than 60")) OR (XB ("55+" OR "55 and over" OR "55 and older" OR "over 55" OR "older than 55")) OR (XB ("50+" OR "50 and over" OR "50 and older" OR "over 50" OR "older than 50")) OR (XB (pensioner*)) OR (XB ("old* person")) OR (XB (late* N2 life)) OR (XB (geriatr*)) OR (XB ("elder*")) OR (XB ("very old")) OR (XB ("oldest old")) OR (XB ("old age")) OR (XB ("older adult*")) OR (XB (senior*)) OR (XB (retired OR retirement OR retiree))) AND ((XB ("aging well")) OR (XB (fulfillment)) OR (XB ("fulfilled life")) OR (XB (meaning* N2 life)) OR (XB ("sense of purpose*")) OR (XB ("optimal aging")) OR (XB (flourish*)) OR (XB (thriving OR thrive*)) OR (XB ("active aging")) OR (XB ("positive aging")) OR (XB ("healthy aging")) OR (XB (happy OR happiness)) OR (XB ("good health")) OR (XB ("mental health")) OR (XB ("cognitive health")) OR (XB ("physical health")) OR (XB ("successful aging")) OR (XB ("life satisfaction")) OR (XB ("quality of life")) OR (XB (wellness)) OR (XB ("well being" OR wellbeing))) AND ((XB ("meta analys*" OR metaanalys* OR "meta synthes*" OR metasynthes*)) OR (XB "systematic review*")) | 2025-09-03T12:48:35.553Z | 1576 |
| **S42** | S41 OR S40 | (XB ("meta analys*" OR metaanalys* OR "meta synthes*" OR metasynthes*)) OR (XB "systematic review*") | 2025-09-03T12:48:26.734Z | 97902 |
| **S41** | XB ("meta analys*" OR metaanalys* OR "meta synthes*" OR metasynthes*) | XB ("meta analys*" OR metaanalys* OR "meta synthes*" OR metasynthes*) | 2025-09-03T12:48:22.726Z | 58924 |
| **S40** | XB "systematic review*" | XB "systematic review*" | 2025-09-03T12:48:19.107Z | 61571 |
| **S39** | S38 OR S37 OR S36 OR S35 OR S34 OR S33 OR S32 OR S31 OR S30 OR S29 OR S28 OR S27 OR S26 OR S25 OR S24 OR S23 OR S22 OR S21 OR S20 OR S19 OR S18 | (XB ("aging well")) OR (XB (fulfillment)) OR (XB ("fulfilled life")) OR (XB (meaning* N2 life)) OR (XB ("sense of purpose*")) OR (XB ("optimal aging")) OR (XB (flourish*)) OR (XB (thriving OR thrive*)) OR (XB ("active aging")) OR (XB ("positive aging")) OR (XB ("healthy aging")) OR (XB (happy OR happiness)) OR (XB ("good health")) OR (XB ("mental health")) OR (XB ("cognitive health")) OR (XB ("physical health")) OR (XB ("successful aging")) OR (XB ("life satisfaction")) OR (XB ("quality of life")) OR (XB (wellness)) OR (XB ("well being" OR wellbeing)) | 2025-09-03T12:48:13.617Z | 544286 |
| **S38** | XB ("aging well") | XB ("aging well") | 2025-09-03T12:48:06.863Z | 265 |
| **S37** | XB (fulfillment) | XB (fulfillment) | 2025-09-03T12:48:01.622Z | 6770 |
| **S36** | XB ("fulfilled life") | XB ("fulfilled life") | 2025-09-03T12:47:57.534Z | 71 |
| **S35** | XB (meaning* N2 life) | XB (meaning* N2 life) | 2025-09-03T12:47:53.740Z | 8809 |
| **S34** | XB ("sense of purpose*") | XB ("sense of purpose*") | 2025-09-03T12:47:49.816Z | 1629 |
| **S33** | XB ("optimal aging") | XB ("optimal aging") | 2025-09-03T12:47:45.697Z | 84 |
| **S32** | XB (flourish*) | XB (flourish*) | 2025-09-03T12:47:41.857Z | 7383 |
| **S31** | XB (thriving OR thrive*) | XB (thriving OR thrive*) | 2025-09-03T12:47:37.995Z | 8759 |
| **S30** | XB ("active aging") | XB ("active aging") | 2025-09-03T12:47:33.957Z | 349 |
| **S29** | XB ("positive aging") | XB ("positive aging") | 2025-09-03T12:47:29.618Z | 246 |
| **S28** | XB ("healthy aging") | XB ("healthy aging") | 2025-09-03T12:47:25.415Z | 3460 |
| **S27** | XB (happy OR happiness) | XB (happy OR happiness) | 2025-09-03T12:47:21.559Z | 31987 |
| **S26** | XB ("good health") | XB ("good health") | 2025-09-03T12:47:15.353Z | 3185 |
| **S25** | XB ("mental health") | XB ("mental health") | 2025-09-03T12:47:11.700Z | 267686 |
| **S24** | XB ("cognitive health") | XB ("cognitive health") | 2025-09-03T12:47:07.964Z | 1449 |
| **S23** | XB ("physical health") | XB ("physical health") | 2025-09-03T12:47:03.700Z | 27635 |
| **S22** | XB ("successful aging") | XB ("successful aging") | 2025-09-03T12:46:58.934Z | 2049 |
| **S21** | XB ("life satisfaction") | XB ("life satisfaction") | 2025-09-03T12:46:54.827Z | 18990 |
| **S20** | XB ("quality of life") | XB ("quality of life") | 2025-09-03T12:46:51.123Z | 95587 |
| **S19** | XB (wellness) | XB (wellness) | 2025-09-03T12:46:47.489Z | 240596 |
| **S18** | XB ("well being" OR wellbeing) | XB ("well being" OR wellbeing) | 2025-09-03T12:46:44.470Z | 240596 |
| **S17** | S16 OR S15 OR S14 OR S13 OR S12 OR S11 OR S10 OR S9 OR S8 OR S7 OR S6 OR S5 OR S4 OR S3 OR S2 OR S1 | (XB (sexagenarian* OR septuagenarian* OR septagenarian* OR octogenarian* OR nonagenarian* OR centenarian* OR centarian* OR supercentenarian* OR supercentarian*)) OR (XB ("65+" OR "65 and over" OR "65 and older" OR "over 65" OR "older than 65")) OR (XB ("60+" OR "60 and over" OR "60 and older" OR "over 60" OR "older than 60")) OR (XB ("55+" OR "55 and over" OR "55 and older" OR "over 55" OR "older than 55")) OR (XB ("50+" OR "50 and over" OR "50 and older" OR "over 50" OR "older than 50")) OR (XB (pensioner*)) OR (XB ("old* person")) OR (XB (late* N2 life)) OR (XB (geriatr*)) OR (XB ("elder*")) OR (XB ("very old")) OR (XB ("oldest old")) OR (XB ("old age")) OR (XB ("older adult*")) OR (XB (senior*)) OR (XB (retired OR retirement OR retiree)) | 2025-09-03T12:46:31.687Z | 386051 |
| **S16** | XB (sexagenarian* OR septuagenarian* OR septagenarian* OR octogenarian* OR nonagenarian* OR centenarian* OR centarian* OR supercentenarian* OR supercentarian*) | XB (sexagenarian* OR septuagenarian* OR septagenarian* OR octogenarian* OR nonagenarian* OR centenarian* OR centarian* OR supercentenarian* OR supercentarian*) | 2025-09-03T12:46:24.809Z | 843 |
| **S15** | XB ("65+" OR "65 and over" OR "65 and older" OR "over 65" OR "older than 65") | XB ("65+" OR "65 and over" OR "65 and older" OR "over 65" OR "older than 65") | 2025-09-03T12:46:20.376Z | 39065 |
| **S14** | XB ("60+" OR "60 and over" OR "60 and older" OR "over 60" OR "older than 60") | XB ("60+" OR "60 and over" OR "60 and older" OR "over 60" OR "older than 60") | 2025-09-03T12:46:14.994Z | 68963 |
| **S13** | XB ("55+" OR "55 and over" OR "55 and older" OR "over 55" OR "older than 55") | XB ("55+" OR "55 and over" OR "55 and older" OR "over 55" OR "older than 55") | 2025-09-03T12:46:10.781Z | 24460 |
| **S12** | XB ("50+" OR "50 and over" OR "50 and older" OR "over 50" OR "older than 50") | XB ("50+" OR "50 and over" OR "50 and older" OR "over 50" OR "older than 50") | 2025-09-03T12:46:06.951Z | 80562 |
| **S11** | XB (pensioner*) | XB (pensioner*) | 2025-09-03T12:46:02.401Z | 284 |
| **S10** | XB ("old* person") | XB ("old* person") | 2025-09-03T12:45:57.670Z | 1232 |
| **S9** | XB (late* N2 life) | XB (late* N2 life) | 2025-09-03T12:45:52.203Z | 24697 |
| **S8** | XB (geriatr*) | XB (geriatr*) | 2025-09-03T12:45:47.148Z | 19009 |
| **S7** | XB ("elder*") | XB ("elder*") | 2025-09-03T12:45:42.590Z | 75154 |
| **S6** | XB ("very old") | XB ("very old") | 2025-09-03T12:45:37.280Z | 1605 |
| **S5** | XB ("oldest old") | XB ("oldest old") | 2025-09-03T12:45:32.160Z | 1447 |
| **S4** | XB ("old age") | XB ("old age") | 2025-09-03T12:45:25.752Z | 14169 |
| **S3** | XB ("older adult*") | XB ("older adult*") | 2025-09-03T12:45:22.595Z | 73013 |
| **S2** | XB (senior*) | XB (senior*) | 2025-09-03T12:45:08.844Z | 33397 |
| **S1** | XB (retired OR retirement OR retiree) | XB (retired OR retirement OR retiree) | 2025-09-03T12:45:03.062Z | 17830 |

## Medline

| 1 | (retired or retirement or retiree).ab,kf,ti. | 30306 |
| --- | --- | --- |
| 2 | senior*.ab,kf,ti. | 59441 |
| 3 | older adult* .ab,kf,ti. | 158226 |
| 4 | old age.ab,kf,ti. | 36516 |
| 5 | oldest old.ab,kf,ti. | 3562 |
| 6 | very old.ab,kf,ti. | 5208 |
| 7 | elder* .ab,kf,ti. | 343387 |
| 8 | geriatr*.ab,kf,ti. | 90419 |
| 9 | (late* adj2 life).ab,kf,ti. | 43712 |
| 10 | old* person .ab,kf,ti. | 2374 |
| 11 | pensioner*.ab,kf,ti. | 1160 |
| 12 | ("50+" or "50 and over" or "50 and older" or "over 50" or "older than 50").ab,kf,ti. | 1668689 |
| 13 | ("60+" or "60 and over" or "60 and older" or "over 60" or "older than 60").ab,kf,ti. | 1128413 |
| 14 | ("65+" or "65 and over" or "65 and older" or "over 65" or "older than 65").ab,kf,ti. | 567611 |
| 15 | ("55+" or "55 and over" or "55 and older" or "over 55" or "older than 55").ab,kf,ti. | 492386 |
| 16 | (sexagenarian* or septuagenarian* or septagenarian* or octogenarian* or nonagenarian* or centenarian* or centarian* or supercentenarian* or supercentarian*).ab,kf,ti. | 9091 |
| 17 | 1 or 2 or 3 or 4 or 5 or 6 or 7 or 8 or 9 or 10 or 11 or 12 or 13 or 14 or 15 or 16 | 3913302 |
| 18 | ("well being" or wellbeing).ab,kf,ti. | 189221 |
| 19 | wellness.ab,kf,ti. | 18382 |
| 20 | quality of life.ab,kf,ti. | 465565 |
| 21 | life satisfaction.ab,kf,ti. | 13613 |
| 22 | successful aging.ab,kf,ti. | 2748 |
| 23 | physical health.ab,kf,ti. | 36696 |
| 24 | cognitive health.ab,kf,ti. | 3203 |
| 25 | mental health.ab,kf,ti. | 287310 |
| 26 | good health.ab,kf,ti. | 12490 |
| 27 | (happy or happiness).ab,kf,ti. | 23143 |
| 28 | healthy aging.ab,kf,ti. | 10159 |
| 29 | positive aging.ab,kf,ti. | 191 |
| 30 | active aging.ab,kf,ti. | 887 |
| 31 | (thriving or thrive*).ab,kf,ti. | 22592 |
| 32 | flourish*.ab,kf,ti. | 7157 |
| 33 | optimal aging.ab,kf,ti. | 79 |
| 34 | sense of purpose* .ab,kf,ti. | 996 |
| 35 | (meaning* adj2 life).ab,kf,ti. | 3805 |
| 36 | fulfilled life.ab,kf,ti. | 36 |
| 37 | fulfillment.ab,kf,ti. | 5998 |
| 38 | aging well.ab,kf,ti. | 265 |
| 39 | 18 or 19 or 20 or 21 or 22 or 23 or 24 or 25 or 26 or 27 or 28 or 29 or 30 or 31 or 32 or 33 or 34 or 35 or 36 or 37 or 38 | 960060 |
| 40 | systematic review* .ab,kf,ti. | 394624 |
| 41 | ("meta analys*" or metaanalys* or "meta synthes*" or metasynthes*).ab,kf,ti. | 357050 |
| 42 | 40 or 41 | 545113 |
| 43 | 17 and 39 and 42 | 8654 |

## Scopus

| (TITLE-ABS-KEY(Retired OR Retirement OR Retiree OR senior* OR "Older adult*" OR "Old age" OR Sexagenarian* OR Septuagenarian* OR Septagenarian* OR Octogenarian* OR Nonagenarian* OR Centenarian* OR Centarian* OR Supercentenarian* OR Supercentarian* OR "Oldest old" OR Senium OR "Very old" OR geriatr* OR (late* W/2 life) OR "old* person" OR pensioner* OR "50+" OR "50 and older" OR "50 and over" OR "50+" or "50 and over" or "50 and older" or "over 50" or "older than 50" OR "60+" or "60 and over" or "60 and older" or "over 60" or "older than 60" OR "65+" or "65 and over" or "65 and older" or "over 65" or "older than 65" OR "55+" or "55 and over" or "55 and older" or "over 55" or "older than 55") AND TITLE-ABS-KEY("Well being" OR Wellbeing OR Wellness OR "Quality of life" OR "Life satisfaction" OR "Successful aging" OR "Physical health" OR "Cognitive health" OR "Mental health" OR "Good health" OR Happiness OR Happy OR "Healthy aging" OR "Positive aging" OR "Active aging" OR Thriving OR thrive* OR Flourishing OR "Optimal aging" OR "Sense of purpose" OR (Meaning* W/2 life) OR "Fulfilled life" OR Fulfillment OR "Aging well") AND TITLE("Systematic review" OR "Meta analys*" OR metaanalys* OR "Meta synthes*" OR metasynthes*)) | 11476 |
| --- | --- |

## Cochrane

| “Well being” OR Wellbeing OR Wellness OR “Quality of life” OR “Life satisfaction” OR “Successful aging” OR “Physical health” OR “Cognitive health” OR “Mental health” OR “Good health” OR Happiness OR Happy OR “Healthy aging” OR “Positive aging” OR “Active aging” OR Thriving OR thrive* OR Flourishing OR “Optimal aging” OR “Sense of purpose” OR (Meaning* NEAR/2 life) OR “Fulfilled life” OR Fulfillment OR “Aging well” in Title Abstract Keyword AND Retired OR Retirement OR Retiree OR senior* OR “Older adult*” OR “Old age” OR Sexagenarian* OR Septuagenarian* OR Septagenarian* OR Octogenarian* OR Nonagenarian* OR Centenarian* OR Centarian* OR Supercentenarian* OR Supercentarian* OR “Oldest old” OR “Very old” OR geriatr* OR (late* NEAR/2 life) OR “old* person” OR pensioner* OR “50 and older” OR “50 and over” or "60 and over" or "60 and older" or "over 60" or "older than 60" OR "65 and over" or "65 and older" or "over 65" or "older than 65" or "55 and over" or "55 and older" or "over 55" or "older than 55" in Title Abstract Keyword - (Word variations have been searched) | 325 |
| --- | --- |
