# Supplementary material for: Factors affecting well-being among retired older adults: A protocol for an umbrella review
Source: PLoS One. 2026 Jun 17;21(6):e0351425. doi: 10.1371/journal.pone.0351425 (PMC13274834; doi:10.1371/journal.pone.0351425)
Supplement: Attachment — Submitted filename: Response to Reviewers.docx [file pone.0351425.s004.docx]

| **Comment** | **Response** |
| --- | --- |
| **Define “umbrella review” more clearly.** The protocol should provide a clearer explanation of what an umbrella review is, how it differs from other forms of evidence synthesis, and why this review type is appropriate for the present research question. | Thank you for this helpful suggestion. We have revised the opening paragraph of the Methods section to define an umbrella review, distinguish it from systematic and scoping reviews, and clarify why this approach is appropriate for the present research question. |
| **Clarify the specific gap this review addresses.** Around lines 111–113, the authors note that older adults’ well-being in retirement is shaped by multiple factors. The manuscript would benefit from a more explicit explanation of why an umbrella review is especially well suited to addressing this gap. In other words, what does this type of review make visible that prior reviews have not? | Thank you. We have revised the final gap paragraph in the Introduction to explain more explicitly what an umbrella review contributes beyond prior single-domain systematic reviews. Specifically, we clarify that an umbrella review can make visible cross-domain patterns, inconsistencies, duplication, and neglected dimensions that may not be apparent when reviews are considered separately. |
| **Clarify the population criteria.** I encourage the authors to provide more stringent and explicit guidelines for the populations included in the review. I understand that the focus is on global aging rather than only population aging in Canada. However, retirement is also an institutional and normative process, and the meaning of “retirement” may vary substantially across national, cultural, and policy contexts. These differences could complicate synthesis if they are not addressed directly. At the same time, describing these differing retirement contexts may itself serve an important descriptive purpose, though this possibility is not yet sufficiently discussed. Either way, I would like to see a more developed explanation of which populations are included or excluded and why. The authors might also consider whether demographic criteria, such as fertility or mortality cutoffs, could help define aging societies or aging populations more systematically. | Thank you for this thoughtful observation. We agree that retirement is shaped by national, institutional, cultural, and policy contexts, and that this variability should be addressed explicitly. We have revised the Population section to clarify that eligibility is based on retirement status and age of the review population, not on whether the study country meets demographic thresholds for an “aging society.” Because the review question focuses on retired older adults globally, applying country-level fertility or mortality cutoffs would risk excluding relevant reviews from settings where retirement is experienced differently but remains relevant to well-being. Instead, we will extract and describe contextual information about retirement definitions, policy settings, and geographic contexts where reported by review authors. |
| **Reconsider the use of “interventions.”** The term “interventions” feels somewhat too narrow or variable-like for the scope of the protocol. I would suggest broadening the language to something like “policy efforts” or “policy and programmatic efforts.” I recognize that there are many cultural, community-based, and grassroots programs designed to support aging individuals in different countries. However, if the authors intend to conduct a cross-national review, these efforts may not be equally visible or documented across contexts. Policies, by contrast, may be more consistently identifiable across countries. | Thank you for this helpful comment. We agree that the term “interventions” may be interpreted narrowly, particularly in a cross-national review where formal policies, community initiatives, cultural practices, and grassroots programs may vary in visibility and documentation across contexts. However, we have retained the term “interventions” in the research question because it is consistent with JBI umbrella review terminology and supports alignment with the review’s eligibility framework. To address the reviewer’s concern, we have revised the **Interventions and Exposures** section to clarify that interventions will be interpreted broadly to include not only formal programs, but also policy, programmatic, community-based, organizational, and other supportive efforts intended to influence wellbeing during or after retirement. |
| **Justify the role of library science expertise in the search strategy.** I am not deeply familiar with library science, but I would like to see more justification for why this expertise is sufficient to refine the search strategy, especially given the protocol’s interest in under-studied topics such as spirituality and cultural contexts of aging. Would the review be strengthened by including a generalist expert in retirement studies alongside a panel of scholars with more specific expertise in cultural and spiritual dimensions of aging? At minimum, it may be helpful for the authors to explain whether the search strategy will be vetted by such experts, particularly since these experts may also be asked to assess the quality of included studies. | Thank you for this important comment. We have revised the Search Strategy section to clarify that the knowledge synthesis librarian contributed expertise in developing, translating, and optimizing database searches, while the broader research team contributed subject-matter expertise in aging, retirement, population health, well-being, and qualitative and mixed-methods research. The search strategy was also peer reviewed by a second librarian using PRESS guidelines, which is a recognized standard for improving the quality of electronic search strategies. We have further clarified that content expertise from the research team informed the conceptual framing of the five well-being domains and will continue to inform screening, extraction, and synthesis. |
