# Supplementary material for: Factors affecting well-being among retired older adults: A protocol for an umbrella review
Source: PLoS One. 2026 Jun 17;21(6):e0351425. doi: 10.1371/journal.pone.0351425 (PMC13274834; doi:10.1371/journal.pone.0351425)
Supplement: Peer review history

# Editor report: Decision Letter 0

**DOI:** 10.1371/journal.pone.0351425.r001

**Authors:** katsuya oi

7 May 2026

PONE-D-26-07750

Factors Affecting Wellbeing Among Retired Older Adults: A Protocol for an Umbrella Review

PLOS One

Dear Dr. Yasin,

Thank you for submitting your manuscript to PLOS ONE. After careful consideration, we feel that it has merit but does not fully meet PLOS ONE’s publication criteria as it currently stands. Therefore, we invite you to submit a revised version of the manuscript that addresses the points raised during the review process.

==============================

Thank you for the opportunity to make an editorial decision on behalf of *PLOS ONE* . Because this submission is a protocol, and in the interest of time, I have made an independent decision to recommend revisions. Please consider the following comments and ask the authors to respond point by point.

My own research on retirement has primarily focused on the U.S. population. I recognize that this protocol is situated in the Canadian context, so while my substantive knowledge is relevant, it may also be somewhat limited in relation to the specific national setting.

I appreciate the overall aim and motivation of the protocol. Retirement is indeed a transition rather than a simple classification, and whether it is experienced as “good” or “bad” often depends on broader social, institutional, cultural, and individual circumstances.

I also find it especially valuable that the protocol includes spiritual well-being. Aging individuals often grapple with mortality while also navigating culturally specific forms of reflection, meaning-making, and adjustment. This dimension seems highly relevant to the study of retirement and later-life well-being.

That said, I offer the following suggestions for revision:

1. **Define “umbrella review” more clearly.**
  The protocol should provide a clearer explanation of what an umbrella review is, how it differs from other forms of evidence synthesis, and why this review type is appropriate for the present research question.
2. **Clarify the specific gap this review addresses.**
  Around lines 111–113, the authors note that older adults’ well-being in retirement is shaped by multiple factors. The manuscript would benefit from a more explicit explanation of why an umbrella review is especially well suited to addressing this gap. In other words, what does this type of review make visible that prior reviews have not?
3. **Clarify the population criteria.**
  I encourage the authors to provide more stringent and explicit guidelines for the populations included in the review. I understand that the focus is on global aging rather than only population aging in Canada. However, retirement is also an institutional and normative process, and the meaning of “retirement” may vary substantially across national, cultural, and policy contexts. These differences could complicate synthesis if they are not addressed directly. At the same time, describing these differing retirement contexts may itself serve an important descriptive purpose, though this possibility is not yet sufficiently discussed. Either way, I would like to see a more developed explanation of which populations are included or excluded and why. The authors might also consider whether demographic criteria, such as fertility or mortality cutoffs, could help define aging societies or aging populations more systematically.
4. **Reconsider the use of “interventions.”**
  The term “interventions” feels somewhat too narrow or variable-like for the scope of the protocol. I would suggest broadening the language to something like “policy efforts” or “policy and programmatic efforts.” I recognize that there are many cultural, community-based, and grassroots programs designed to support aging individuals in different countries. However, if the authors intend to conduct a cross-national review, these efforts may not be equally visible or documented across contexts. Policies, by contrast, may be more consistently identifiable across countries.
5. **Justify the role of library science expertise in the search strategy.**
  I am not deeply familiar with library science, but I would like to see more justification for why this expertise is sufficient to refine the search strategy, especially given the protocol’s interest in under-studied topics such as spirituality and cultural contexts of aging. Would the review be strengthened by including a generalist expert in retirement studies alongside a panel of scholars with more specific expertise in cultural and spiritual dimensions of aging? At minimum, it may be helpful for the authors to explain whether the search strategy will be vetted by such experts, particularly since these experts may also be asked to assess the quality of included studies.

==============================

Please submit your revised manuscript by Jun 21 2026 11:59PM. If you will need more time than this to complete your revisions, please reply to this message or contact the journal office at plosone@plos.org. When you're ready to submit your revision, log on to https://www.editorialmanager.com/pone/ and select the 'Submissions Needing Revision' folder to locate your manuscript file.

Please include the following items when submitting your revised manuscript:

- A letter that responds to each point raised by the academic editor and reviewer(s). You should upload this letter as a separate file labeled 'Response to Reviewers'.
- A marked-up copy of your manuscript that highlights changes made to the original version. You should upload this as a separate file labeled 'Revised Manuscript with Track Changes'.
- An unmarked version of your revised paper without tracked changes. You should upload this as a separate file labeled 'Manuscript'.

If you would like to make changes to your financial disclosure, please include your updated statement in your cover letter. Guidelines for resubmitting your figure files are available below the reviewer comments at the end of this letter.

If applicable, we recommend that you deposit your laboratory protocols in protocols.io to enhance the reproducibility of your results. Protocols.io assigns your protocol its own identifier (DOI) so that it can be cited independently in the future. For instructions see: https://journals.plos.org/plosone/s/submission-guidelines#loc-laboratory-protocols. Additionally, PLOS ONE offers an option for publishing peer-reviewed Lab Protocol articles, which describe protocols hosted on protocols.io. Read more information on sharing protocols at https://plos.org/protocols?utm_medium=editorial-email&utm_source=authorletters&utm_campaign=protocols.

As the corresponding author, your ORCID iD is verified in the submission system and will appear in the published article. PLOS supports the use of ORCID, and we encourage all coauthors to register for an ORCID iD and use it as well. Please encourage your coauthors to verify their ORCID iD within the submission system before final acceptance, as unverified ORCID iDs will not appear in the published article. *Only*  the individual author can complete the verification step; PLOS staff *cannot*  verify ORCID iDs on behalf of authors.

We look forward to receiving your revised manuscript.

Kind regards,

katsuya oi, PhD

Academic Editor

PLOS One

Journal Requirements:

When submitting your revision, we need you to address these additional requirements.

1.Please ensure that your manuscript meets PLOS ONE's style requirements, including those for file naming. The PLOS ONE style templates can be found at

https://journals.plos.org/plosone/s/file?id=wjVg/PLOSOne_formatting_sample_main_body.pdf and

https://journals.plos.org/plosone/s/file?id=ba62/PLOSOne_formatting_sample_title_authors_affiliations.pdf

2. Thank you for stating the following financial disclosure:

“Y.M.Y: received funding from Social Sciences and Humanities Research Council.

Funding Opportunity: Insight Development Grants

Application Number: 430-2025-00006”

Please state what role the funders took in the study.  If the funders had no role, please state: "The funders had no role in study design, data collection and analysis, decision to publish, or preparation of the manuscript."

If this statement is not correct you must amend it as needed.

Please include this amended Role of Funder statement in your cover letter; we will change the online submission form on your behalf.

3. Please provide a complete Data Availability Statement in the submission form, ensuring you include all necessary access information or a reason for why you are unable to make your data freely accessible. If your research concerns only data provided within your submission, please write "All data are in the manuscript and/or supporting information files" as your Data Availability Statement.

4. When completing the data availability statement of the submission form, you indicated that you will make your data available on acceptance. We strongly recommend all authors decide on a data sharing plan before acceptance, as the process can be lengthy and hold up publication timelines. Please note that, though access restrictions are acceptable now, your entire data will need to be made freely accessible if your manuscript is accepted for publication. This policy applies to all data except where public deposition would breach compliance with the protocol approved by your research ethics board. If you are unable to adhere to our open data policy, please kindly revise your statement to explain your reasoning and we will seek the editor's input on an exemption. Please be assured that, once you have provided your new statement, the assessment of your exemption will not hold up the peer review process.

5. Please include captions for your Supporting Information files at the end of your manuscript, and update any in-text citations to match accordingly. Please see our Supporting Information guidelines for more information: http://journals.plos.org/plosone/s/supporting-information.

6. If the reviewer comments include a recommendation to cite specific previously published works, please review and evaluate these publications to determine whether they are relevant and should be cited. There is no requirement to cite these works unless the editor has indicated otherwise.

7. Please review your reference list to ensure that it is complete and correct. If you have cited papers that have been retracted, please include the rationale for doing so in the manuscript text, or remove these references and replace them with relevant current references. Any changes to the reference list should be mentioned in the rebuttal letter that accompanies your revised manuscript. If you need to cite a retracted article, indicate the article’s retracted status in the References list and also include a citation and full reference for the retraction notice.

Additional Editor Comments:

Thank you for the opportunity to make an editorial decision on behalf of PLOS ONE. Because this submission is a protocol, and in the interest of time, I have made an independent decision to recommend revisions. Please consider the following comments and ask the authors to respond point by point.

My own research on retirement has primarily focused on the U.S. population. I recognize that this protocol is situated in the Canadian context, so while my substantive knowledge is relevant, it may also be somewhat limited in relation to the specific national setting.

I appreciate the overall aim and motivation of the protocol. Retirement is indeed a transition rather than a simple classification, and whether it is experienced as “good” or “bad” often depends on broader social, institutional, cultural, and individual circumstances.

I also find it especially valuable that the protocol includes spiritual well-being. Aging individuals often grapple with mortality while also navigating culturally specific forms of reflection, meaning-making, and adjustment. This dimension seems highly relevant to the study of retirement and later-life well-being.

That said, I offer the following suggestions for revision:

Define “umbrella review” more clearly.

The protocol should provide a clearer explanation of what an umbrella review is, how it differs from other forms of evidence synthesis, and why this review type is appropriate for the present research question.

Clarify the specific gap this review addresses.

Around lines 111–113, the authors note that older adults’ well-being in retirement is shaped by multiple factors. The manuscript would benefit from a more explicit explanation of why an umbrella review is especially well suited to addressing this gap. In other words, what does this type of review make visible that prior reviews have not?

Clarify the population criteria.

I encourage the authors to provide more stringent and explicit guidelines for the populations included in the review. I understand that the focus is on global aging rather than only population aging in Canada. However, retirement is also an institutional and normative process, and the meaning of “retirement” may vary substantially across national, cultural, and policy contexts. These differences could complicate synthesis if they are not addressed directly. At the same time, describing these differing retirement contexts may itself serve an important descriptive purpose, though this possibility is not yet sufficiently discussed. Either way, I would like to see a more developed explanation of which populations are included or excluded and why. The authors might also consider whether demographic criteria, such as fertility or mortality cutoffs, could help define aging societies or aging populations more systematically.

Reconsider the use of “interventions.”

The term “interventions” feels somewhat too narrow or variable-like for the scope of the protocol. I would suggest broadening the language to something like “policy efforts” or “policy and programmatic efforts.” I recognize that there are many cultural, community-based, and grassroots programs designed to support aging individuals in different countries. However, if the authors intend to conduct a cross-national review, these efforts may not be equally visible or documented across contexts. Policies, by contrast, may be more consistently identifiable across countries.

Justify the role of library science expertise in the search strategy.

I am not deeply familiar with library science, but I would like to see more justification for why this expertise is sufficient to refine the search strategy, especially given the protocol’s interest in under-studied topics such as spirituality and cultural contexts of aging. Would the review be strengthened by including a generalist expert in retirement studies alongside a panel of scholars with more specific expertise in cultural and spiritual dimensions of aging? At minimum, it may be helpful for the authors to explain whether the search strategy will be vetted by such experts, particularly since these experts may also be asked to assess the quality of included studies.

[Note: HTML markup is below. Please do not edit.]

[NOTE: If reviewer comments were submitted as an attachment file, they will be attached to this email and accessible via the submission site. Please log into your account, locate the manuscript record, and check for the action link "View Attachments". If this link does not appear, there are no attachment files.]

To ensure your figures meet our technical requirements, please review our figure guidelines: https://journals.plos.org/plosone/s/figures

You may also use PLOS’s free figure tool, NAAS, to help you prepare publication quality figures: https://journals.plos.org/plosone/s/figures#loc-tools-for-figure-preparation.

NAAS will assess whether your figures meet our technical requirements by comparing each figure against our figure specifications.

# Author comment: Author response to Decision Letter 1

**DOI:** 10.1371/journal.pone.0351425.r002

10 May 2026

See attached "Respond to Reviewers" file.

Attachment Submitted filename: Response to Reviewers.docx

# Editor report: Decision Letter 1

**DOI:** 10.1371/journal.pone.0351425.r003

**Authors:** Xuexing Luo

27 May 2026

Factors Affecting Well-being Among Retired Older Adults: A Protocol for an Umbrella Review

PONE-D-26-07750R1

Dear Dr. Yasin,

We’re pleased to inform you that your manuscript has been judged scientifically suitable for publication and will be formally accepted for publication once it meets all outstanding technical requirements.

Within one week, you’ll receive an e-mail detailing the required amendments. When these have been addressed, you’ll receive a formal acceptance letter and your manuscript will be scheduled for publication.

An invoice will be generated when your article is formally accepted. Please note, if your institution has a publishing partnership with PLOS and your article meets the relevant criteria, all or part of your publication costs will be covered. Please make sure your user information is up-to-date by logging into Editorial Manager at Editorial Manager® and clicking the ‘Update My Information' link at the top of the page. For questions related to billing, please contact billing support.

If your institution or institutions have a press office, please notify them about your upcoming paper to help maximize its impact. If they’ll be preparing press materials, please inform our press team as soon as possible -- no later than 48 hours after receiving the formal acceptance. Your manuscript will remain under strict press embargo until 2 pm Eastern Time on the date of publication. For more information, please contact onepress@plos.org.

Kind regards,

Xuexing Luo

Academic Editor

PLOS One

Additional Editor Comments (optional):

Reviewers' comments:

# Editor report: Acceptance letter

**DOI:** 10.1371/journal.pone.0351425.r004

**Authors:** Xuexing Luo

PONE-D-26-07750R1

PLOS One

Dear Dr. Yasin,

I'm pleased to inform you that your manuscript has been deemed suitable for publication in PLOS One. Congratulations! Your manuscript is now being handed over to our production team.

At this stage, our production department will prepare your paper for publication. This includes ensuring the following:

* All references, tables, and figures are properly cited

* All relevant supporting information is included in the manuscript submission,

* There are no issues that prevent the paper from being properly typeset

You will receive further instructions from the production team, including instructions on how to review your proof when it is ready. Please keep in mind that we are working through a large volume of accepted articles, so please give us a few days to review your paper and let you know the next and final steps.

Lastly, if your institution or institutions have a press office, please let them know about your upcoming paper now to help maximize its impact. If they'll be preparing press materials, please inform our press team within the next 48 hours. Your manuscript will remain under strict press embargo until 2 pm Eastern Time on the date of publication. For more information, please contact onepress@plos.org.

You will receive an invoice from PLOS for your publication fee after your manuscript has reached the completed accept phase. If you receive an email requesting payment before acceptance or for any other service, this may be a phishing scheme. Learn how to identify phishing emails and protect your accounts at https://explore.plos.org/phishing.

If we can help with anything else, please email us at customercare@plos.org.

Thank you for submitting your work to PLOS ONE and supporting open access.

Kind regards,

PLOS ONE Editorial Office Staff

on behalf of

Dr. Xuexing Luo

Academic Editor

PLOS One
